# Effectiveness of a reduced dose of efavirenz plus 2 NRTIs as maintenance antiretroviral therapy with the guidance of therapeutic drug monitoring

**DOI:** 10.7448/IAS.17.4.19524

**Published:** 2014-11-02

**Authors:** Shang-Ping Yang, Wen-Chun Liu, Kuan-Yeh Lee, Bing-Ru Wu, Yi-Ching Su, Pei-Ying Wu, Jun-Yu Zhang, Yu-Zhen Luo, Hsin-Yun Sun, Sui-Yuan Chang, Shu-Wen Lin, Chien-Ching Hung

**Affiliations:** 1 Center for Infection Control, National Taiwan University Hospital, Taipei City, Taiwan; 2 Internal Medicine, National Taiwan University Hospital, Taipei City, Taiwan; 3 Internal Medicine, National Taiwan University Hospital Hsinchu Branch, Hsin-Chu city, Taiwan; 4 Clinical Laboratory Sciences and Medical Biotechno, National Taiwan University College of Medicine, Taipei City, Taiwan; 5 Graduate Institute of Clinical Pharmacy, College of Medicine, National Taiwan University, Taipei City, Taiwan

## Abstract

**Introduction:**

Wide inter-patient variation of plasma efavirenz (EFV) concentrations has been observed, and a substantial proportion of HIV-positive patients may have unnecessarily higher plasma EFV concentrations than recommended while receiving EFV-containing combination antiretroviral therapy (cART) at the currently recommended daily dose of 600 mg. A lower daily dose (400 mg) of EFV has recently been demonstrated to be as efficacious as the recommended 600 mg when combined with tenofovir/mtricitabine in a multinational clinical trial, with a lower incidence of adverse effects. We aimed to use a therapeutic drug monitoring (TDM)-guided strategy to optimize the EFV dose in HIV-positive Taiwanese patients.

**Materials and Methods:**

The plasma EFV concentrations at 12 hours (C12) after taking the previous dose were determined among HIV-positive adults who had received EFV-containing cART with viral suppression (plasma HIV RNA load (PVL) <200 copies/mL). For those with EFV C12 >2.0 mg/L, EFV (Stocrit, MSD) was reduced to half a tablet daily. Determinations of EFV C12 were repeated 4–12 weeks after switch using high-performance liquid chromatography. CYP2B6 G516T polymorphisms were determined using polymerase-chain-reaction restriction fragment-length polymorphism.

**Results:**

Between April 2013 and June 2014, 111 patients (95.5% male; mean age, 39 years; 96.4% with PVL <40 copies/ml; 26.4% HBsAg-positive and 7.5% anti-HCV-positive) with plasma C12 efavirenz >2.0 mg/L were switched to a reduced dose (1/2# hs) of EFV; 45.5% of them had CYP2B6 G516T or TT genotypes; and 32.4% weighed 60 kg or less. The mean baseline EFV C12 before switch was 3.65 mg/L (interquartile range (IQR), 2.62–4.17) for 111 patients, which decreased to 1.96 mg/L (IQR, 1.53–2.33) for 64 patients who had completed follow-up of C12 EFV 4 weeks after switch, with a reduction of 49.4% (IQR, 38.9–57.0%). As of 10 July, 2014, all of the 38 patients (100%) who had completed at least one follow-up of PVL achieved undetectable PVL (<40 copies/ml) following switch to a reduced dose of EFV after a mean observation of 13 weeks (IQR, 7–15 weeks).

**Conclusions:**

Switch to cART containing a half tablet of EFV (1/2#) in HIV-positive Taiwanese patients with higher plasma EFV concentrations who had achieved viral suppression could maintain successful viral suppression with the guidance of TDM.

